# Extract from a Letter to the Obstetric Gazette from Dr. Bigelow, at Berlin

**Published:** 1885-10

**Authors:** 


### Extract from a Letter to the Obstetric Gazette from Dr. Bigelow, at Berlin.

Writing from Berlin to the *Obstetric Gazette*, Dr. Bigelow says: "Dr. Martin's polyclinic furnishes him with an exceptionally large material, and, during five weeks just gone by, he has completed considerably over one hundred operations. His statistics are creeping up to those of Schroeder, whom he may soon pass, and his results are extremely good. With such large experience, with such daily association with grave disease, and with such facility of technique, it is not surprising that success should follow him. He arrives at a diagnosis speedily and almost faultlessly, and operates rapidly and with signal coolness. Dr. Duoclius, himself a surgeon of reputation, is his first assistant; another administers the anæsthetic; while another, in conjunction with an intelligent matron, handles the instruments, sponges, etc. Dr. Martin sits between the knees of his patient, and the two assistants are also seated. The galvanized iron operating-table, invented by the matron, has an arrangement by which its middle third can be dropped, thus facilitating the placing of the dressings. Every one looks comfortable, and I am quite impressed with the advantages of this plan. The first assistant sits at the left of the patient, manipulates the abdominal parietes, keeps the intestines out of the cavity, covering them with a hot towel, and secures all bleeding points. The operations in abdominal surgery are all made in a special room, devoted exclusively to this class of work. It is literally saturated with carbolic spray. All the water used is boiled, and everything and everybody are made as aseptic as possible. The hair of the patient is shaved from the pubes, the abdomen is washed with brown soap and water, then with bichloride solution, and then with lemon juice. The temperature of the room is kept well up. The tumor being removed, and the stump being sewn up, the left hand is passed into the cavity behind the stump to the posterior cal-de-sac; the right hand then pushes a long pair of forceps through and into the vagina; an ordinary rubber drainage tube is then pulled through into the peritoneal cavity and left in situ. The other end drains into the vagina. Not altogether a safe proceeding, it seems to me; but where the results are so good, it is hypercriticism to find fault."

In a letter to the New Orleans *Medical and Surgical Journal*, Dr. Chassaignac reports the lesions found post-mortem in a ent dying after a punctured wound of chest, death being by syncope, six days after the reception of the injury.

"Post-mortem examination showed that the weapon, after going through the lung, had penetrated the pericardium, and inflicted a wound about three-fourths of an inch long and one-eighth of an inch deep in the wall of the right ventricle. The edges of this cut were granulating, and seemed already to have slightly adhered. In addition, the pleural cavity contained a large quantity of blood, the lung was carnified, and the pericardium held nearly an ounce of pus."

The relation that this incision bore to the direction of the muscular fibres is not stated, but is important; as wounds of the heart, as a rule, are immediately fatal, especially when of large size, or transverse to the muscular fibres of the ventricles, which then gape and allow hemorrhage into the pericardium. When the wound is parallel to the fibres of the ventricle, death may be delayed, as the spiral arrangement of the different layers of fibres has a tendency to occlude the wound. Though recovery has taken place after wounds of the auricles, such injuries are more fatal than those of the ventricles, on account of the absence of this spiral arrangement of their muscular fibres and the thinness of their walls.

